# Clinical characteristics of leukemic optic nerve infiltration in pediatric acute lymphoblastic leukemia: 3 cases and a literature review

**DOI:** 10.3389/fmed.2026.1786820

**Published:** 2026-03-18

**Authors:** Yiwen Li, Guohong Tian, Aishwarya Sriram, Ling Qin, Quangang Xu, Fang Lei, Cheng Zhang

**Affiliations:** 1 Department of Ophthalmology, The First Affiliated Hospital, and College of Clinical Medicine of Henan University of Science and Technology, Luoyang, China; 2 Department of Ophthalmology, Eye, Ear, Nose and Throat Hospital of Fudan University, Shanghai, China; 3 Moran Eye Center, University of Utah, Salt Lake City, UT, United States; 4 Department of Hematology, The First Affiliated Hospital, and College of Clinical Medicine of Henan University of Science and Technology, Luoyang, China; 5 Senior Department of Ophthalmology, The Third Medical Center of PLA General Hospital, Beijing, China

**Keywords:** CNS involvement, multimodal therapy, neuro-ophthalmic manifestations, optic nerve infiltration, pediatric acute lymphoblastic leukemia

## Abstract

Pediatric optic nerve infiltration in acute lymphoblastic leukemia (ALL) is a rare yet critical neuro-ophthalmic emergency, often signaling central nervous system (CNS) involvement or relapse. In this case series we present three cases of optic nerve infiltration in pediatric patients with ALL. The patients underwent systemic chemotherapy, intrathecal chemotherapy, and, in some instances, orbital radiation therapy, leading to varying degrees of visual recovery. In addition to presenting these cases, we review the existing literature and discuss the pathogenesis, clinical features, diagnostic approaches, imaging findings, and treatment strategies for this condition. Based on our cases and prior reports, we outline a practical diagnostic and treatment framework that prioritizes early recognition to reduce the risk of irreversible vision loss. For relapse or refractory disease, intensified therapy may be considered, including chimeric antigen receptor CD19 CAR T-cell therapy and hematopoietic stem cell transplantation. Optic nerve infiltration in ALL requires prompt, coordinated management among hematology, oncology, and ophthalmology specialists. Timely and appropriately intensified treatment may improve visual outcomes and survival.

## 1 Introduction

Acute lymphoblastic leukemia (ALL) is a heterogeneous hematologic malignancy characterized by the abnormal proliferation of immature lymphocytes in the bone marrow, peripheral blood, and other organs. ALL can arise from B-cell precursors (approximately 88% of cases) or T-cell precursors (1). It is the most common malignant tumor in children, accounting for about 25% of all childhood cancers, with a peak incidence around 3 years of age (2). Although predominantly a childhood disease, ALL can also occur in adults, with increasing incidence in individuals over 50 (3).

Approximately 25%–40% of patients with ALL experience ocular involvement (4–6) which can be classified into two categories based on etiology. Primary ocular manifestations include anterior segment uveitis, orbital infiltration, and neuro-ophthalmic manifestations associated with central nervous system (CNS) leukemia, such as optic nerve infiltration and cranial nerve involvement. Secondary ocular manifestations can also occur, due to hematologic abnormalities (such as anemia, thrombocytopenia, and hyperviscosity syndrome) or adverse effects of treatment (such as corticosteroids, chemotherapy, hematopoietic stem cell transplantation, radiation, and immunosuppressive therapy) (5, 7).

Clinical retrospective studies have shown that optic nerve infiltration accounts for 15%–25% of ocular manifestations in leukemia and is strongly correlated with CNS leukemia (6). Optic nerve infiltration in ALL is often regarded as a sign of CNS relapse, as it may cause rapid and irreversible vision loss, making it a neuro-oncological emergency (8). In particular, isolated optic nerve infiltration is frequently misdiagnosed as optic neuritis or periorbital optic neuritis, leading to treatment delays and potentially unnecessary interventions (9).

Due to the rarity of this condition, standardized diagnostic and treatment guidelines are lacking, and clinical management largely relies on previously published case reports. In this article, we present three pediatric cases of acute lymphoblastic leukemia with optic nerve infiltration and provide an extensive review of the literature. We propose the clinical course and available follow-up assessments, including visual acuity and multimodal optic nerve evaluation combining optical coherence tomography (OCT) measurements of retinal nerve fiber layer (RNFL) thickness and ganglion cell complex/ganglion cell-inner plexiform layer (GCC/GCIPL) with serial contrast-enhanced MRI to assess optic nerve and optic nerve sheath involvement. By integrating our cases with the published literature, we summarize a multidisciplinary, stepwise decision support approach to diagnosis and treatment escalation, with particular attention to the timing and indications for radiotherapy. We also address relapse and refractory disease planning and discuss CAR T-cell therapy and HSCT as systemic disease-control or consolidation options after CNS or optic nerve stabilization.

## 2 Case presentation

**FIGURE 1:**
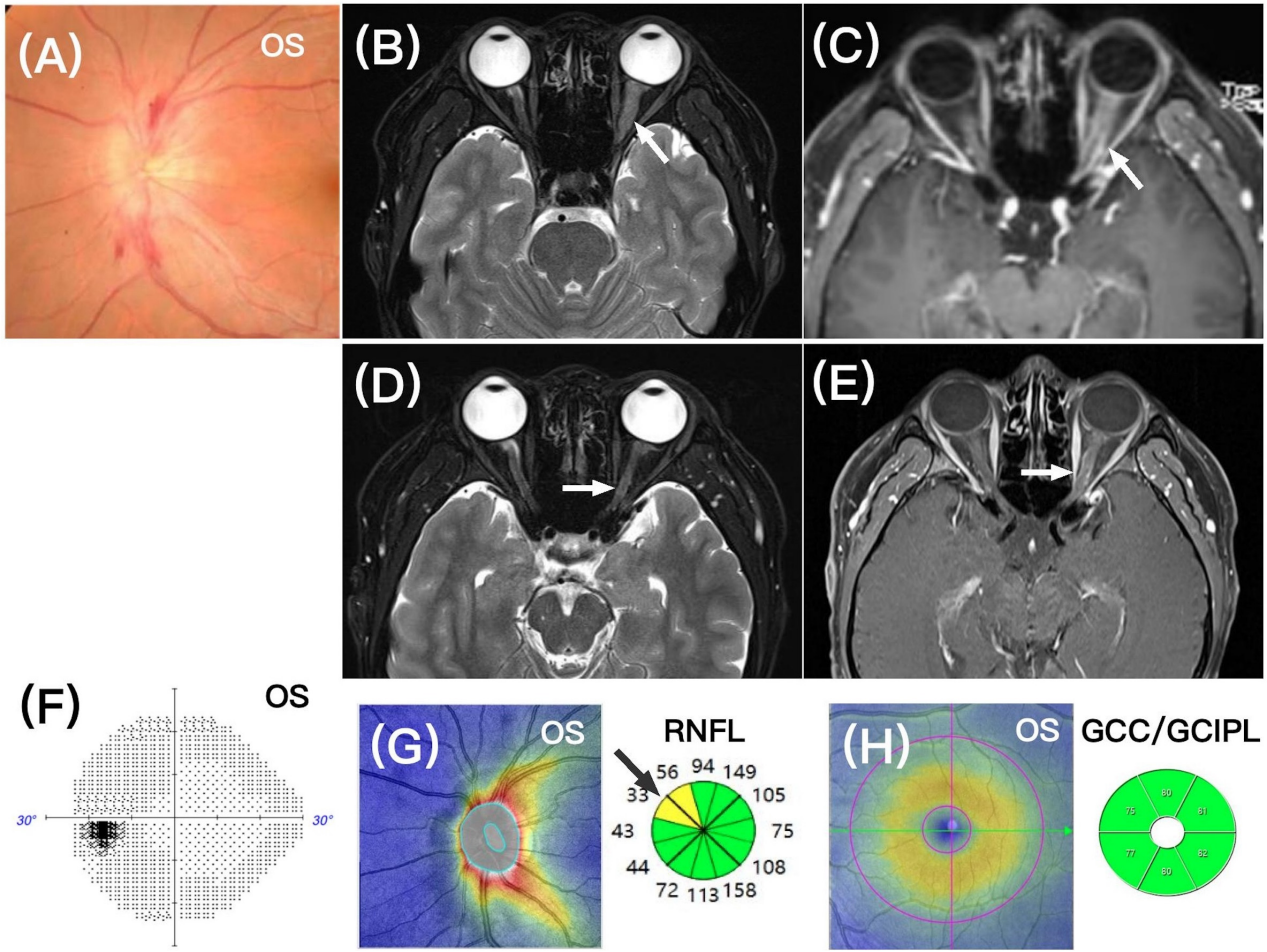
**(A)** Fundus photograph showing mild optic disc elevation in the left eye with blurred borders and surrounding hemorrhages. **(B,C)** Orbital MRI showing thickening of the left optic nerve in the orbital segment, with enhancement of the nerve sheath, respectively (white arrows). **(D,E)** One month after treatment, orbital MRI showed improvement in optic nerve thickening and reduced enhancement, respectively (white arrows). **(F)** One year follow-up visual field (HVF) test showed normal findings in the left eye. **(G,H)** One-year follow-up optical coherence tomography (OCT) showed very mild focal thinning of the peripapillary retinal nerve fiber layer (RNFL) in the left eye (black arrow, sectors in yellow color), while the ganglion cell complex/ganglion cell-inner plexiform layer (GCC/GCIPL) appeared normal.

Case 1: a 17-years-old male presented with 3 days of blurry vision in the left eye and pain in the left temporal region. He had been diagnosed with B-ALL 5 years ago and was receiving regular maintenance therapy with azathioprine and methotrexate. Three months prior, he underwent radiotherapy for left knee joint relapse, achieving complete remission. His best-corrected visual acuity (BCVA) was 20/20 in both eyes. A very mild relative afferent pupillary defect (RAPD) was present in the left eye. Fundusoscopic examination revealed left optic disc swelling with surrounding hemorrhages (Figure 1A). Humphrey visual field testing (HVF) showed an enlarged physiological blind spot in the left eye. An orbital MRI showed enlargement of the left optic nerve and enhancement of the optic nerve sheath (Figures 1B, C). The brain MRI showed no abnormalities, and the right eye was normal. Cerebrospinal fluid (CSF) analysis via lumbar puncture was unremarkable. There were no other signs of relapse elsewhere in the body.

He received the systemic “Hyper-CVAD” chemotherapy regimen along with intrathecal injection of methotrexate, cytarabine, and dexamethasone. One month later, the left temporal pain resolved, and BCVA remained 20/20 in both eyes. The left optic disc swelling decreased, and the HVF showed some improvement in the enlarged physiological blind spot. Orbital MRI demonstrated resolution of optic nerve thickening with less enhancement (Figures 1D, E). Follow-up 1 year later showed complete recovery of the visual field in the left eye (Figure 1F). However, OCT revealed mild focal thinning of the left RNFL (Figure 1G), and the average RNFL thickness was notably thinner compared to the unaffected right eye (right eye: 105 μm, left eye: 87 μm). The OCT-GCC/GCIPL appeared unaffected (Figure 1H). The patient has been followed for 18 months after receiving CD19 CAR T-cell therapy, and his condition has stabilized.

**FIGURE 2:**
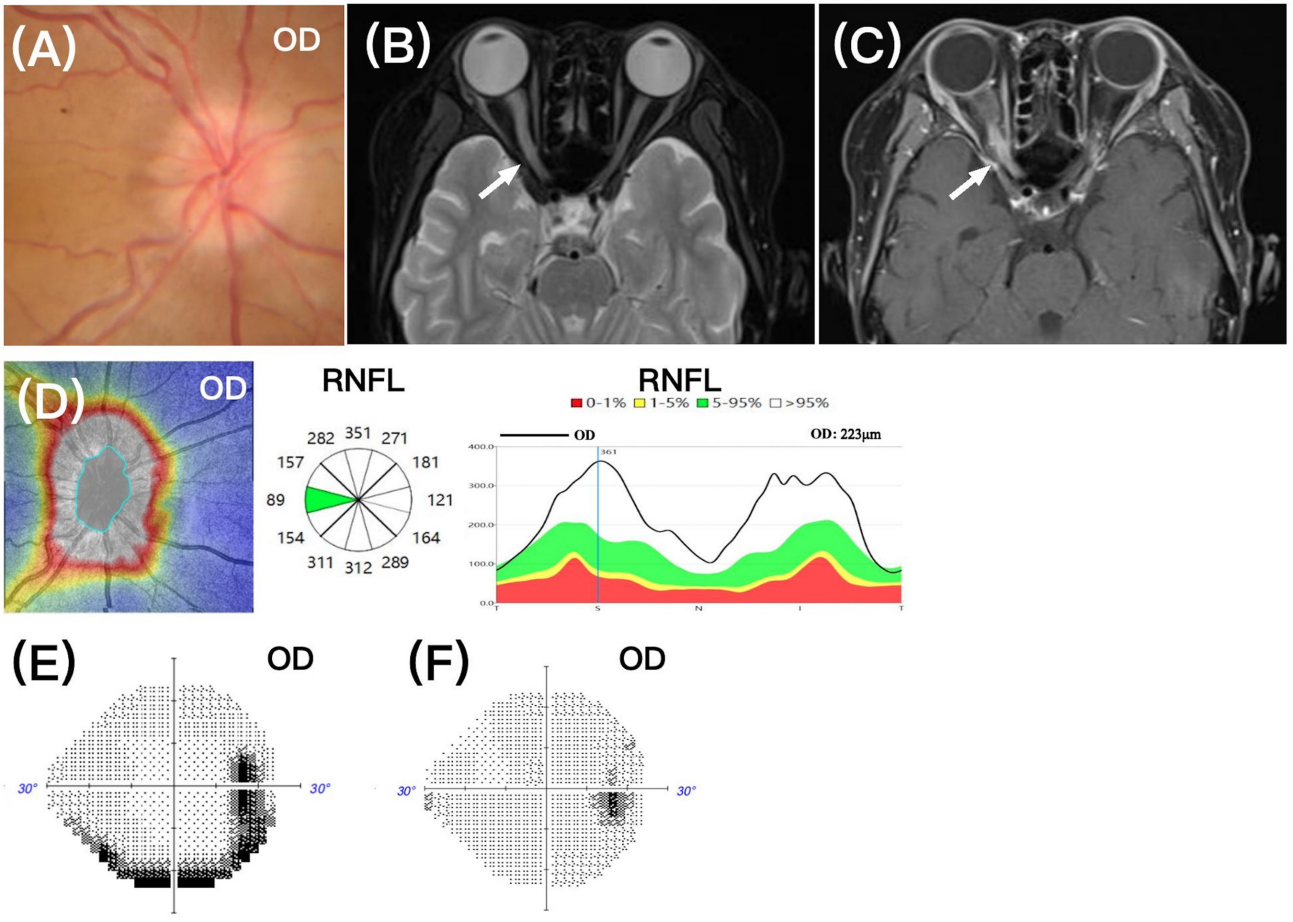
**(A)** Fundus photograph showing optic disc elevation in the right eye with blurred margins. **(B,C)** Orbital MRI showing thickening of the right optic nerve in the orbital segment with a long T2 signal, and enhancement in the posterior segment of the right optic nerve, respectively (white arrows). **(D)** OCT-RNFL scan (middle panel) showing increased thickness in the right eye (average thickness 223 μm), consistent with right optic disc edema. **(E,F)** Twenty days after treatment HVF showed an inferior peripheral rim/arcuate defect in the right eye, and 10 months after treatment the HVF appeared unremarkable in the right eye.

Case 2: a 15-years-old male presented with 1 day of blurred vision in the right eye. He had been diagnosed with T-ALL 7 months earlier and was undergoing regular treatment with the “Hyper-CVAD” regimen, and he was currently in complete remission. On examination, BCVA was no light perception in the right eye and 20/20 in the left eye. The right optic disc showed swelling (Figure 2A), and orbital MRI revealed thickening of the right optic nerve, with a long T2 signal, and enhancement of the posterior segment of the right optic nerve (Figures 2B, C). OCT-RNFL measured 223 μm in the right eye (Figure 2D), and the left eye was completely normal. Brain MRI showed no abnormalities, and CSF analysis via lumbar puncture was unremarkable. There were no other signs of relapse elsewhere in the body.

In addition to the “Hyper-CVAD” chemotherapy regimen, the patient received radiation therapy to the right orbit, with 10 sessions totaling 20 Gy, and intrathecal chemotherapy with methotrexate, cytarabine, and dexamethasone. After 20 days, BCVA in the right eye improved to 20/20, and the right optic disc swelling partially subsided. The HVF test showed an inferior peripheral rim/arcuate defect in the right eye (Figure 2E), which had fully recovered after 10 months (Figure 2F).

**FIGURE 3:**
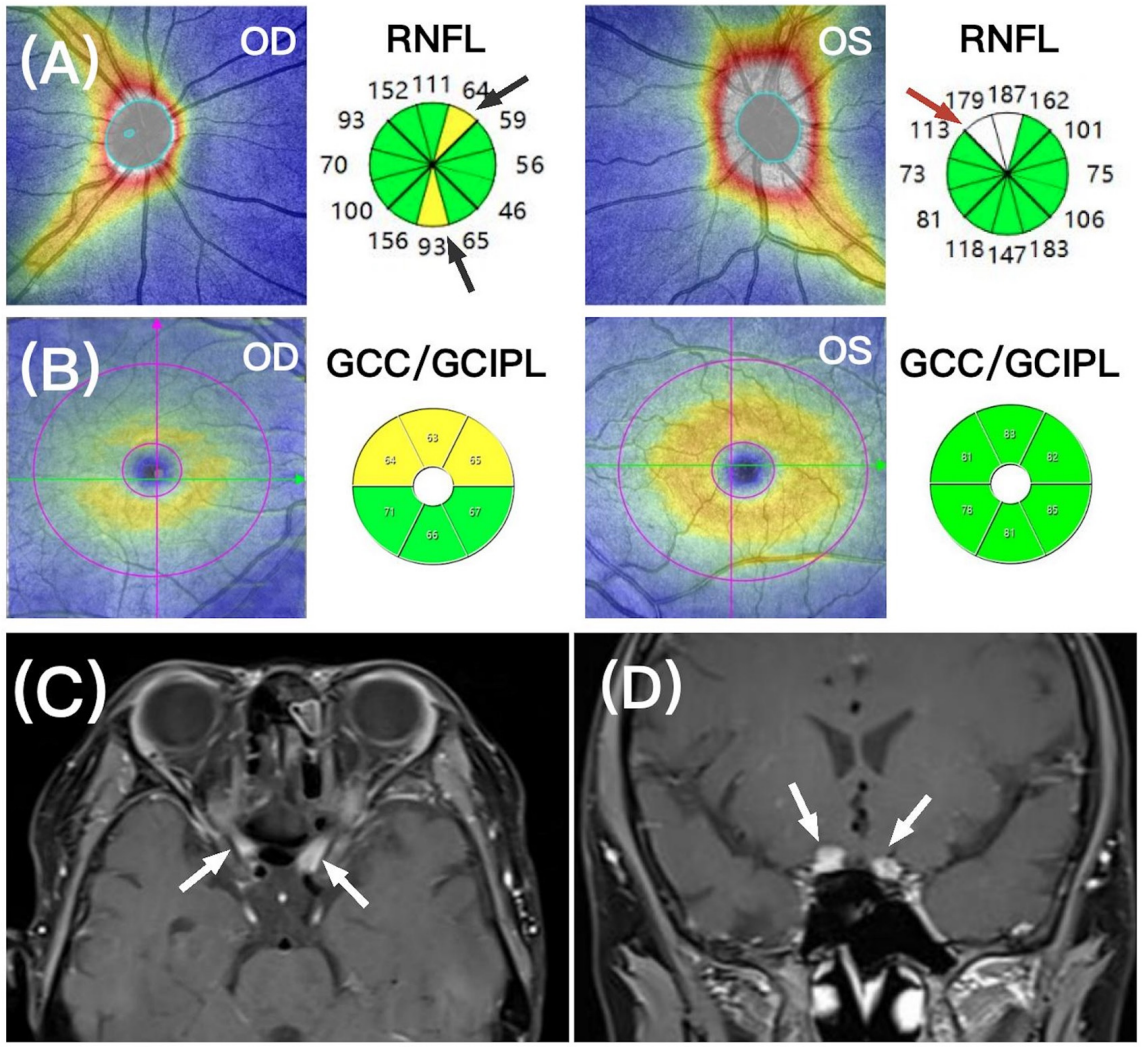
**(A)** Recurrence at 1 year after treatment, OCT-RNFL (top panel) showed focal mild thinning (black arrows, sectors in yellow color) of the right optic nerve, and mild edema in the left optic disc (red arrows, sectors in white color). **(B)** OCT-GCC (middle panel) showed superior thinning of the right eye, indicating partial optic atrophy. The left eye showed no GCC/GCIPL thinning. **(C,D)** Orbital MRI showed bilateral prechiasmatic optic nerve thickening and enhancement (white arrows).

Unfortunately, 1 year after treatment, the patient’s vision declined again, becoming no light perception in the right eye and 20/100 in the left eye. Fundus examination revealed pallor of the right optic disc, and no significant swelling in either eye. OCT-RNFL showed mild focal thinning in the right eye, consistent with partial atrophy due to prior optic nerve infiltration, with mild edema in the left eye due to new onset (Figure 3A). OCT-GCC showed superior thinning in the right eye, also consistent with prior optic nerve damage, with no thinning in the left eye (Figure 3B). Orbital MRI showed bilateral prechiasmatic optic nerve thickening and enhancement (Figures 3C, D). Due to bone marrow suppression and uncontrollable sepsis caused by *Pseudomonas aeruginosa* and *Escherichia coli* after repeated systemic chemotherapy, the patient ultimately discontinued treatment.

**FIGURE 4:**
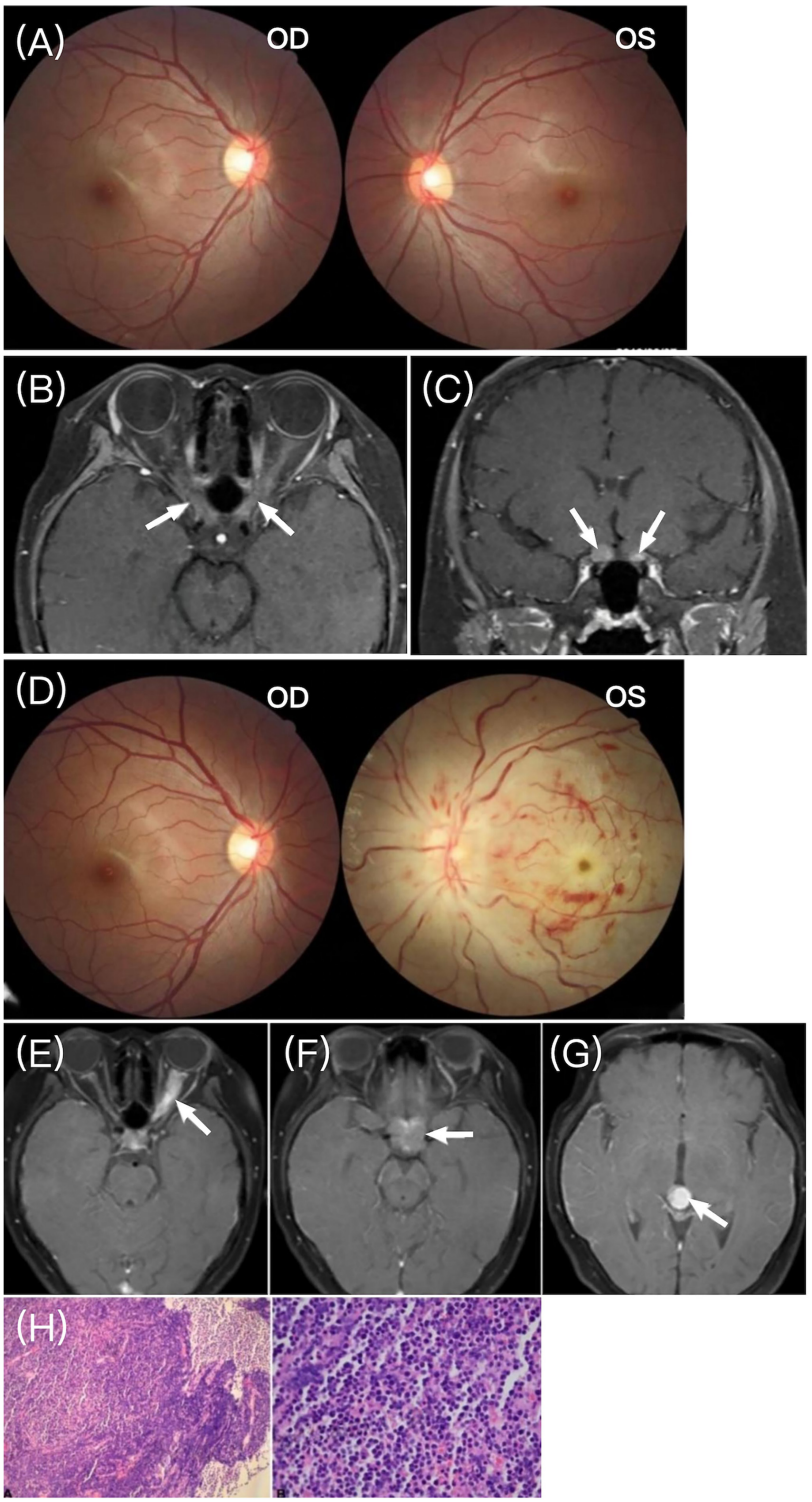
**(A)** Fundus photograph showing no abnormalities in either eye upon presentation. **(B,C)** Orbital MRI showing thickening and enhancement of the optic nerves anterior to the optic chiasm in both eyes (white arrows). **(D)** Fundus photographs showing severe optic disc edema in the left eye, with tortuous blood vessels, retinal hemorrhage, and a cherry-red spot in the macula; the right eye was normal. **(E–G)** Cranial MRI showing thickening and enhancement of the left optic nerve, abnormal enhancement in the sellar region, and a round-like enhancing lesion in the fourth ventricle, respectively (white arrows). **(H)** Histopathological study of left optic nerve biopsy with immunohistochemistry (not shown) indicating B-ALL.

Case 3: a 12-years-old girl presented with progressive vision loss in both eyes for more than 1 month. She had no significant medical history. The BCVA was 20/100 in the right eye and 20/400 in the left eye. Funduscopic examination was normal in both eyes (Figure 4A). Orbital MRI showed thickening and enhancement of the optic nerves near the optic chiasm (Figures 4B, C), leading to a diagnosis of bilateral optic neuritis. The patient’s parents refused lumbar puncture and CSF examination. After receiving intravenous methylprednisolone, her vision slightly improved.

However, 20 days later, the patient developed severe left eye pain. BCVA was 20/100 in the right eye and no light perception in the left. The left optic disc showed severe swelling, and the retina exhibited signs of central retinal artery occlusion (CRAO), with tortuous blood vessels, hemorrhages, and a cherry-red spot in the macula (Figure 4D). Brain MRI revealed thickening and enhancement of the left optic nerve, abnormal enhancement in the sellar region, and a round enhancing lesion in the fourth ventricle (Figures 4E–G). A left optic nerve biopsy was performed, and immunohistochemistry confirmed B-ALL (Figure 4H). The patient was subsequently referred to the hematology department, where B-ALL was diagnosed, and systemic treatment was initiated. The patient has since been lost to follow-up.

## 3 Discussion

To contextualize this rare manifestation of pediatric ALL, we conducted a targeted literature search and review of previously published case reports. We searched PubMed, Web of Science Core Collection, and Google Scholar for reports published between January 1, 2005 and October 31, 2025 using combinations of terms related to ALL and optic nerve involvement (e.g., “acute lymphoblastic leukemia/ALL” and “optic nerve/optic neuropathy/optic nerve infiltration/leukemic optic neuropathy”). Titles, abstracts, and full texts were screened for relevance. We included pediatric (≤18 years) ALL cases with clinical and/or radiologic evidence of optic nerve infiltration and extractable individual-level data on clinical course and outcomes. Among these reported cases, 5 were B-ALL, 12 were T-ALL, and 6 were unspecified, with ages ranging from 2 to 18 years. Clinical data from our cases and previously published studies were analyzed, including the study year, gender, age, leukemia type, timing of optic nerve infiltration, affected eye, ocular findings, other relapse sites, CSF results, neuro-imaging findings, treatment regimen, and post-treatment visual acuity and outcomes. The cases reported in our study are summarized in Table 1, and previous studies are shown in Tables 2–4.

**TABLE 1:** Optic nerve infiltration in ALL (our cases).

Case	Gender/ age	Leukemia type	Time of remission to optic nerve infiltration	Affected eye	BCVA	Ocular findings	CSF	Imaging	Treatment regimen	Post-treatment visual acuity	Outcome
1	M/17	B-ALL	3 months	OS	20/20	Optic disc(+)	(−)	MRI:ON(+) Enhancement(+)	IVc, IT	VF recovery	CAR T-cell therapy, remained stable for 18 months
2	M/15	T-ALL	1 month	OD	NLP	Optic disc(+)	(−)	MRI:ON(+) Enhancement(+)	IVc, IT, XRT(Orbital 20Gy)	Vision recovery	Relapsed again after 1 year
			Recurrence	OU	OD CF OS 20/100	Optic disc(+)	NA	MRI:ON(+) Enhancement(+)	IVc	No improvement in vision	Treatment stopped
3	F/12	B-ALL	Initial onset	OU	OD 20/100 OS 20/400	Normal fundus	NA	MRI:ON(+) Enhancement(+)	IVs	Short-term improvement	Progression occurred 20 days later
			Progression	OU	OD 20/100 OS NLP	Optic disc(+) Retinal(+)	NA	MRI:Cranial + ON(+) Cranial + ON Enhancement(+)	NA	NA	Lost to follow-up

**TABLE 2:** Published pediatric studies of optic nerve infiltration in B-ALL.

References	Gender/ age	Leukemia type	Time of remission to optic nerve infiltration	Affected eye	Ocular findings	Other relapse sites	CSF	Imaging	Treatment regimen	Post-treatment visual acuity	Outcome
(61)	M/17	B-ALL	7 months	OD	Optic disc(+) Retinal(+)	Bone marrow	(−)	MRI:ON(+)	IVc	Worsening	NA
(62)	M/9	Pre B-ALL	3 years	OD	Optic disc(+)	(−)	(+)	CT:ON(+)	IVc, XRT (Cranial 20Gy)	Vision improvement	Stable condition after 6 months
(63)	F/9	B-ALL	NA	OU	Optic disc(+) Retinal(+)	Cranial	(+)	MRI:Cranial + ON(+) Cranial Enhancement(+)	IVc, XRT(Cranial + Orbital)	Reduced papilledema	NA
(22)	F/7	B-ALL	2 months	OS	Optic disc(+) Retinal(+)	(−)	(+)	MRI:ON(+) Enhancement(+)	IVc, IT, XRT	No improvement in vision	Under follow-up
(64)	M/2	Pre B-ALL	NA	OU	Optic disc(+) Retinal(+)	(−)	(+)	MRI:ON(+) Enhancement(+)	IVc, IT, XRT (Cranial + Orbital 24Gy)	No improvement in vision	Death after 6 months

**TABLE 3:** Published pediatric studies of optic nerve infiltration in T-ALL.

References	Gender/ age	Leukemia type	Time of remission to optic nerve infiltration	Affected eye	Ocular findings	Other relapse sites	CSF	Imaging	Treatment regimen	Post-treatment visual acuity	Outcome
(43)	M/18	T-ALL	NA	OS	Optic disc(+)	(−)	(+)	MRI:ON(+)	IVc, XRT(Cranial 18Gy + Orbital 6Gy)	No improvement in vision	NA
(65)	M/18	T-ALL	6 months	OU	Optic disc(+) Retinal(+)	(−)	(−)	MRI:ON(−)	IVc, IT	No improvement in vision	Death after a few months
(36)	M/16	T-ALL	NA	OU	Optic disc(+)	(−)	(+)	MRI:ON(+)	IVc, IT	Vision improvement	Death after 4 months
(66)	M/15	T-ALL	NA	OS	Optic disc(+) Retinal(+)	Bone marrow, Testicular	(+)	NA	IVc, IT	No improvement in vision	Marrow remission
(45)	M/14	T-ALL	12 months	OD	Optic disc(+) Retinal(+)	(−)	(−)	MRI:ON(+) Enhancement(+)	IVc, XRT(Orbital 24Gy)	Vision improvement	Complete remission maintained after HSCT
(67)	F/14	T-ALL	NA	OS	Optic disc(+) Retinal(+)	(−)	(−)	MRI:ON(+) Enhancement(+)	IVc, IT	Vision improvement	NA
(37)	M/13	T-ALL	8 months	OU	Optic disc(+) Retinal(+)	Cranial	(−)	MRI:ON(+)	IVc, IT	Vision improvement	NA
(15)	F/12	T-ALL	2 months	OU	Optic disc(+) Retinal(+)	Cranial	NA	CT(−), Cranial MRI(+),ON MRI(+), Enhancement(+)	IT	Worsening	Death after 10 months
(8)	M/11	T-ALL	16 months	OS	Optic disc(+) Retinal(+)	(−)	(−)	MRI:ON(+) Enhancement(+)	IVs	Vision recovery	Recurrence after 3 weeks, death
(68)	M/8	T-ALL	NA	OD	Optic disc(+) Retinal(+)	(−)	(−)	CT(−), MRI(−)	XRT(Cranial + Orbital)	No improvement in vision, fundus improvement	NA
(38)	F/7	T-ALL	8 months	OU	Optic disc(+) Retinal(+)	(−)	(−)	MRI:ON(+) Enhancement(+)	IVc, IT, XRT (TBI 12Gy + Orbital 9Gy)	Vision improvement	Remission after HSCT after 3 months
(69)	F/Teen	T-ALL	Initial onset	OU	Optic disc(+) Retinal(+)	(−)	NA	NA	IVc, IVT-MTX	Vision improvement	NA

**TABLE 4:** Published pediatric studies of optic nerve infiltration in unspecified ALL subtype.

References	Gender/ age	Leukemia type	Time of remission to optic nerve infiltration	Affected eye	Ocular findings	Other relapse sites	CSF	Imaging	Treatment regimen	Post-treatment visual acuity	Outcome
(70)	M/15	ALL	7 months	OD	Optic disc(+)	(−)	(+)	MRI:ON(+) Enhancement(+)	IVc	Vision improvement	No recurrence after 6 months
(71)	M/14	ALL	NA	OU	Optic disc(+)	(−)	NA	MRI:ON(+) Enhancement(+)	IVc, IT, XRT (Cranial + Orbital 20Gy)	Vision improvement	6 months: Disease remission
(44)	M/14	ALL	6 months	OU	Optic disc(+) Retinal(+)	Bone marrow, Systemic	(+)	MRI:ON(+) Enhancement(+)	IVc, IT, XRT(Orbital 30Gy)	Vision improvement	Death after 15 months
(72)	M/9	ALL	NA	OU	Optic disc(+) Retinal(+)	(−)	(−)	MRI:ON(+)	IVc, IT, XRT	No improvement in vision	NA
(73)	F/Teen	ALL	NA	OS	Optic disc(+) Retinal(+)	(−)	NA	MRI:ON(+)	XRT(Orbital 18Gy)	Fundus improvement	NA
(74)	Teen	ALL	NA	OD	Optic disc(+)	(−)	NA	CT:ON(+)	IVc, XRT	Vision improvement	NA

M, male; F, female; ALL, acute lymphoblastic leukemia; B-ALL, B-cell acute lymphoblastic leukemia; Pre B-ALL, precursor B-cell acute lymphoblastic leukemia; T-ALL, T-cell acute lymphoblastic leukemia; OD, right eye; OS, left eye; OU, both eyes; IVc, intravenous chemotherapy; IVs, intravenous solution; IVT, intravitreal injection; IT, intrathecal; XRT, external radiation therapy; TBI, total body irradiation; IVT, intravitreal injection; MTX, methotrexate; NA, not available.

### 3.1 Clinical presentation

Optic nerve infiltration in ALL can present either unilaterally or bilaterally. Among previously reported pediatric cases, 12 were unilateral and 11 were bilateral. Of the bilateral cases, both eyes could be affected either simultaneously or sequentially. Most patients experienced acute or subacute vision loss. Of these cases, 6 cases presented with isolated optic nerve infiltration, while 17 had retinal involvement (3 cases with retinal infiltration and 14 with retinal vascular changes and other lesions).

Clinical presentations varied significantly among patients, likely due to the location of tumor infiltration within the optic nerve. When the prelaminar optic nerve is involved, the optic disc can have a creamy or grayish-white elevation, blurred borders, congestion, and edema, with flame- or dot-shaped hemorrhages, hard exudates, and cotton wool spots around the disk (10, 11). When the orbital segment of optic nerve or optic nerve sheath is involved, the degree of optic disc edema is generally less severe, or there may be no obvious optic disc edema. In cases involving the intracanalicular or intracranial segments of the optic nerve, fundus examination may be unremarkable without signs of local infiltration (12).

Irrespective of the infiltration in the prelaminar or orbital segment, retinal involvement may also occur. In previously reported cases, most patients showed retinal venous engorgement, dilation, and hemorrhage. Retinal vascular lesions are relatively common in optic nerve infiltration in ALL, such as venous occlusion. In severe cases, CRAO and exudative retinal detachment may occur. This mechanism may be related to hypercoagulable state of the blood, leukemia cell infiltration, encasement, or the formation of tumor emboli within the blood vessels (13, 14).

Outside of vision loss, patients may present with other symptoms. In our Case 1, the patient experienced temporal pain, while Case 3 presented with ocular pain and pain on eye movement. These symptoms are presumably related to tumor invasion/compression and surrounding inflammatory reaction. Some patients in previous reports have also experienced headaches, which may be associated with leukemia involvement of the meninges or increased intracranial pressure (15). Among previously reported cases of optic nerve infiltration in pediatric ALL, 3 cases were associated with central nervous system relapse. Additionally, 3 reports described bone marrow relapse with systemic features including fever and splenomegaly, and 1 case even reported testicular infiltration.

Regarding timing of presentation, optic nerve infiltration or CNS relapse may occur at any stage of ALL. In our small case series, Cases 1 and 2 achieved disease control and remission before CNS relapse, while Case 3 presented with optic nerve infiltration as the initial finding of ALL. Of the previously reported cases, only one case had optic nerve infiltration as first presentation, while the others were all due to relapses. Eleven previous cases reported the time from remission to relapse, ranging from 2 months to 3 years, with 9 cases relapsing between 2 and 12 months. In Johnson et al.’s report, the majority of patients (81.8%) were also in remission before optic nerve relapse (16).

### 3.2 Etiology and pathogenesis

Johnson et al. (16) reported 10 cases of optic nerve infiltration in pediatric ALL, with 7 cases of B-ALL and 3 cases of T-ALL. In contrast, previous reports showed a higher number of T-ALL cases than B-ALL cases. Advances in contemporary medicine and effective CNS prophylaxis combined with systemic therapy have significantly prolonged survival in patients with ALL. In both adults and children, the overall rate of CNS relapse has been declining (17, 18).

However, Kassar et al. (9) found that leukemic optic nerve infiltration is associated with patients’ survival time. Additionally, even after multiple chemotherapy regimens and prophylactic craniospinal irradiation, optic nerve infiltration may still occur (16). The primary reason for this is that the optic nerve is considered an important “pharmacological sanctuary” for leukemia cells. The blood-brain barrier limits the penetration of chemotherapy drugs, and the anatomical structure of the optic nerve sheath also obstructs the diffusion of intrathecal chemotherapy agents to the retrobulbar optic nerve. As a result, many systemic chemotherapy drugs (even intrathecal route) do not reach effective concentrations in the optic nerve (19). Preventive brain irradiation may miss part of the optic nerve due to ocular shielding (15). While systemic treatment extends the patient’s survival, previously suppressed but not eradicated residual cells have a longer window to cause delayed relapse.

Additionally, optic nerve infiltration may possibly occur through three other pathways: (1) the meningeal or cerebrospinal fluid route, where leukemia cells spread along the optic nerve sheath to the eye through the subarachnoid space (8); (2) hematogenous dissemination, where leukemia cells enter the optic nerve capillary system via the bloodstream, causing optic disc swelling or hemorrhage, often associated with systemic leukemia activity; and (3) direct tissue spread, where leukemia cells infiltrate the optic nerve from the orbit or choroid (20). Therefore, optic nerve infiltration may occur at any stage of ALL.

### 3.3 Cerebrospinal fluid evaluation

While CSF cytology is a routinely used and widely accepted method for diagnosing CNS leukemia, its sensitivity is limited to around 50% due to the fragility of leukemia cells and delays in processing (21). A review by Myers et al. reported that the detection of tumor cells on CSF cytology was positive in approximately 64% of all different types of leukemia cases with optic nerve infiltration (8). In Johnson et al.’s report, the CSF positivity rate was only 36% (16). Among previously reported pediatric ALL cases, 18 patients underwent CSF cytology: 9 were positive and 9 were negative. The positivity rate in these reports was 50% (9/18 cases), which was close to 64% reported by Myers et al., indicating the need for further clinical investigation.

Negative CSF cytology does not reliably exclude optic nerve or CNS involvement. Particularly when clinical findings and neuro imaging are highly suggestive, in suspected cases, MRI may demonstrate optic nerve involvement, such as enhancement of the optic nerve or the optic nerve sheath. In such cases, repeat lumbar puncture for CSF sampling may be considered to reduce false negative results (22, 23). Beyond conventional cytology, CSF flow cytometry may increase the detection rate by approximately two to three fold (24, 25). In addition, advanced CSF molecular approaches have shown feasibility for detecting occult CNS involvement in hematologic malignancies. These include CSF mRNA expression profiling, targeted sequencing of circulating tumor DNA, and high throughput proteomic biomarker profiling (26–28). Although these CSF molecular methods are still in the experimental or clinical trial phase, published studies have shown that CSF ctDNA detection in hematologic malignancies with CNS involvement has a significantly higher detection rate compared to traditional methods (29), highlighting the tremendous potential of these new approaches in identifying occult central nervous system diseases. Therefore, because CSF cytology has limited sensitivity, diagnosis should rely on a comprehensive approach that integrates clinical findings, MRI, and adjunct CSF testing when needed.

### 3.4 Neuro-imaging findings

MRI is the imaging modality of choice for evaluating the brain and orbital soft tissue, such as the globe and optic nerve. Optic nerve infiltration in ALL typically appears on MRI as thickening and enhancement of the optic nerve parenchyma or its sheath, which may be similar to the findings seen in optic neuritis or perineuritis. In particular, when isolated optic nerve involvement is the only initial manifestation in patients without a prior history of ALL, it may be easily confused with inflammatory lesions (9), such as in our Case 3. In previous case reports, only 2 cases showed negative orbital MRI results. Some previous case reports have shown the involvement of the orbital fat tissue, though the extraocular muscles are usually unaffected (8).

PET/CT combines the metabolic imaging with the anatomic localization of CT, yielding high sensitivity for detecting and localizing extramedullary relapse. Beyond identifying relapse at a suspected site, whole body scanning may reveal additional, otherwise overlooked foci. On PET/CT, extramedullary relapse in ALL typically appears as hypermetabolic lesions. In cases of optic nerve infiltration, increased uptake may be seen in the orbital region (30). Although PET/CT is not routinely used in pediatric ALL because of safety considerations, it may provide added diagnostic value in selected scenarios, such as suspected extramedullary relapse or in diagnostically challenging cases (29).

### 3.5 Pathologic examination

When MRI and CSF results are inconclusive, invasive procedures are typically required. Although optic nerve biopsy carries significant risks, it should be considered in patients with blindness or very poor vision to obtain a tissue diagnosis for further treatment (31). Immunohistochemistry may demonstrate positive expression of leukemia associated markers, such as CD10, TdT, and CD20 in optic nerve tissue (32).

### 3.6 Diagnosis and differential diagnosis

In our report, Case 1 and Case 2 were diagnosed and treated early, leading to significant recovery of vision. However, in Case 3, the diagnosis was delayed, resulting in rapid disease progression and eventually CRAO, with a poor prognosis. In patients with a history of ALL, any visual abnormalitiy should prompt an immediate ophthalmologic examination. If there are neuro-ophthalmologic symptoms, MRI assessment is necessary, and optic nerve infiltration must be considered.

When optic nerve involvement is the initial manifestation of ALL (as in Case 3), misdiagnosis as inflammatory optic neuropathy is not uncommon, especially when there is no evidence of CNS or systemic involvement. Age is also an important factor, particularly in young children, as visual symptoms may be overlooked. Early diagnosis is critical to prevent irreversible optic nerve damage and treatment delays. Moreover, in pediatric ALL, optic nerve dysfunction may not be only caused by direct leukemic infiltration. Other mechanisms may contribute, including immune mediated injury and vascular related pathways (33, 34). On MRI, optic nerve thickening and enhancement can overlap between leukemic infiltration and related optic neuritis. In this situation, imaging findings should be interpreted together with CSF studies and the clinical course. A detailed history and careful examination are essential for differential diagnosis. When MRI findings and complete ophthalmologic evaluation remain equivocal and diagnostic uncertainty persists, tissue confirmation may be considered, if clinically feasible, to distinguish leukemic infiltration from optic neuritis or other causes.

### 3.7 Treatment

**FIGURE 5:**
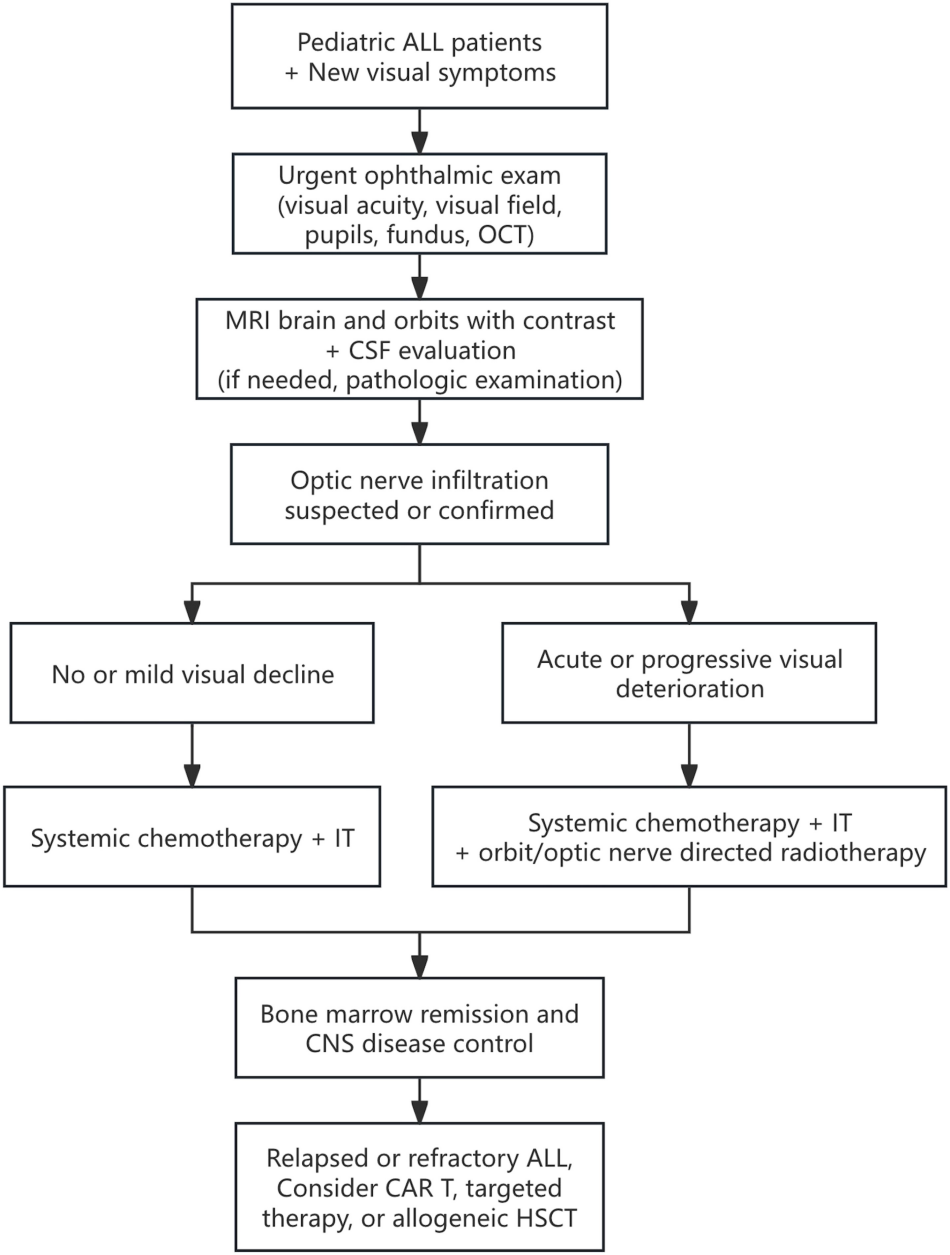
Proposed stepwise clinical approach for suspected optic nerve involvement in pediatric ALL. This algorithm summarizes a decision support pathway based on evidence from published cases. Diagnostic and escalation decisions should be individualized and made in a multidisciplinary setting.

Optic nerve infiltration in ALL is considered a neuro-oncological emergency, and treatment typically requires an aggressive and specialized approach to prevent further deterioration. Banerjee et al. (35) suggested a 7-days window as the maximum threshold for starting chemotherapy to achieve favorable outcomes. Based on our cases and the published literature, we propose a stepwise clinical approach summarized in Figure 5.

Intrathecal (IT) chemotherapy together with systemic chemotherapy remains the primary management strategy reported in the literature in CNS involvement (5, 31). According to the NCCN 2024 Acute Lymphoblastic Leukemia Guidelines (NCCN Clinical Practice Guidelines in Oncology – Acute Lymphoblastic Leukemia, Version 2.2024), intrathecal methotrexate or a combination regimen (methotrexate + cytarabine + steroid) is recommended, with injections given weekly or biweekly (36). Most previous case reports used the combination of intrathecal regimen of methotrexate + cytarabine + dexamethasone/hydrocortisone (9, 37–39). In our study, both Case 1 and Case 2 received this intrathecal chemotherapy regimen. In children with CNS relapse, early intensification of therapy directed at the CNS is essential, including escalation to high-dose chemotherapy and the use of regimens that penetrate the blood–brain barrier, such as high-dose methotrexate (40).

Leukemic cells are highly radiosensitive; thus orbital irradiation may reduce the leukemic burden within the optic nerve sheath, improve CSF flow, and enhance the penetration of intrathecal agents (41), even with low radiation doses (42). If local radiation therapy is initiated early in patients with ON invasion and visual decrease and combined with chemotherapy/IT, it may improve vision and control the disease. While standardized guidelines for orbit/optic nerve directed radiation in leukemic optic neuropathy are lacking, retrospective case series data suggest that radiotherapy is frequently delivered on an emergent basis in vision threatening cases due to ON involvement. In a pediatric case series by Johnson et al., 8/11 patients received emergent local radiation within approximately 1.5 ± 1.2 days of the initial ophthalmologic examination (16). In many previously reported cases, the orbital/optic nerve radiation dose ranged from 6 to 30 Gy (43–45). In our Case 2, the orbital radiation dose was 20 Gy, and the patient’s visual acuity improved markedly and quickly, which was consistent with previous reports.

Radiation-induced optic neuropathy (RION) is the most severe complication of cranial and orbital radiation therapy. Clinical data show that under conventional fractionation (1.8–2 Gy per fraction), RION is rare when the maximum point dose is kept at ≤55 Gy, but the risk increases significantly when doses reach ≥60 Gy. When the lesions near the visual pathway are treated with single or few high-dose fractions (Stereotactic radiosurgery/Fractionated stereotactic radiosurgery, SRS/fSRS), previous experience and retrospective data suggest limiting the maximum point dose to the visual pathway (optic nerve/chiasm) (46).

Emerging treatments, such as intravitreal injections of methotrexate, dexamethasone, and anti-VEGF agents, have been reported to treat ocular infiltration in ALL (including optic nerve infiltration), but large-scale studies validating their efficacy are still lacking (7).

After effective control and remission of disease through CNS-directed therapy (systemic chemotherapy + intrathecal therapy + radiotherapy), patients with relapsed or refractory ALL may consider CAR T-cell therapy, targeted therapies, and allogeneic hematopoietic stem cell transplantation (HSCT) to achieve durable event-free survival. CAR T-cell therapy, particularly products directed against CD19, is an immunotherapy approved by the FDA that engineers a patient’s own T cells to recognize and kill cancer cells (47). Immuno-targeted therapy combats cancer cells by inhibiting specific molecular targets and is often combined with conventional chemotherapy, offering greater selectivity and lower toxicity. B-cell-targeted therapy has made significant advances, particularly against targets such as BCR-ABL, CD19, and CD22 (48). Allogeneic HSCT is also effective in B-ALL, especially for high-risk or relapsed disease (49). By contrast, due to the more complex biology of T-ALL, CAR T-cell and targeted approaches remain largely investigational, with uncertain efficacy and clinical applicability (50, 51). HSCT outcomes in T-ALL are generally less favorable than in B-ALL; nevertheless, HSCT is still considered for high-risk patients and those with CNS relapse (49).

Systemic corticosteroid therapy is an essential treatment for ALL, as it may help alleviate inflammation related to infiltration. It is noteworthy that in our Case 3, the child initially presented with ocular symptoms and was misdiagnosed with optic nerve demyelination. After receiving systemic corticosteroid treatment, vision improved modestly, but relapse and progression occurred once the steroids were stopped. A similar case was previously reported by Myers et al. (8). In malignant hematologic conditions, corticosteroids, due to their immunosuppressive effects, often mask clinical signs of inflammation and infection, and may even alter imaging and pathological findings, leading to false-negative results and further delaying diagnosis (52). The timing of corticosteroid treatment is a challenging decision that must be analyzed based on the individual clinical situation.

### 3.8 Prognosis

Banerjee et al. (35) pointed out that early treatment can significantly improve vision, with optic nerve changes spontaneously resolving within 2–4 weeks after the start of treatment. Similarly, in previously reported cases of optic nerve infiltration in pediatric ALL, most patients showed improvement in vision after treatment, with only two cases worsening due to difficult to control intramedullary relapse. Because of retinal vascular changes or optic nerve atrophy, some cases showed improvement in the fundus appearance but no improvement in the visual acuity. Even with timely treatment, optic nerve infiltration often leads to secondary optic atrophy after resolution, as observed in Cases 1 and 2, as well as in prior studies. Furthermore, although rare, poor prognosis may occur due to RION. However, because the radiation dose is relatively low, the severity is usually not significant (53).

Despite treatment, relapses can also occur. In our Case 2, optic nerve infiltration recurred 1 year after treatment. This may be attributed to the higher overall relapse rate of T-ALL compared to B-ALL (approximately 20%–40%), and its increased tendency for extramedullary relapse, particularly in the CNS and mediastinum (54). High-risk subtypes (such as Ph+ and specific genetic mutations) are often more difficult to control, leading to a higher risk of relapse. Therefore, patients with high relapse risk in ALL should undergo more frequent follow-up after treatment.

Myers et al. (8) reported that the mean survival after a diagnosis of optic nerve invasion in patients with ALL is only 11 months. Among previously reported pediatric ALL cases with available follow-up, 6 of 11 patients (6/11) died, with survival times ranging from 3 weeks to 15 months. Of these, there was 1 death in B-ALL (1/3) and 4 deaths in T-ALL (4/6). Regardless of lineage (T-ALL or B-ALL), prognosis after extramedullary relapse is worse than in patients who never relapse. Notably, the prognosis of isolated CNS relapse is better than relapses that are accompanied by bone marrow relapse (55, 56). Because T-ALL has a greater burden of high-risk biology/complications, relapse and mortality rates are generally higher in T-ALL than in B-ALL (54, 57).

For pediatric ALL patients with optic nerve infiltration, early reduction of leukemic burden in the optic nerve is crucial for improving visual prognosis and prolonging survival. Compared to cases with poor visual prognosis, patients with favorable prognostic features tend to have longer survival (58). If detected early and treated aggressively, incorporating CAR T-cell and immune-targeted therapies alongside allogeneic transplantation may achieve long-term remission and even cure. In Case 1 of our study (B-ALL), complete remission was achieved after CAR T-cell therapy and maintained at the 18 months follow-up, with the disease remaining stable. Previous reports also described two patients who achieved complete remission after HSCT.

Given that relapse is a major determinant of prognosis, improved risk stratification and more sensitive tools for detecting occult CNS disease may further refine follow up strategies and systemic management. Molecular risk stratification in ALL relies on minimal residual disease (MRD) assessment and recurrent genetic subtypes. These features may correlate with treatment resistance and relapse risk, and may also help identify patients with a higher likelihood of CNS or extramedullary disease (59). Sensitive CSF based molecular testing, such as ctDNA or transcriptomic or proteomic profiling, could complement cytology in detecting occult CNS involvement and may support longitudinal monitoring of the neuro ophthalmic course (60). Evidence linking specific molecular features to optic nerve involvement is still limited and warrants further study.

## 4 Conclusion

Previous reports described an ocular involvement rate of approximately 25%–40% in ALL, and optic nerve involvement accounts for 15%–25% of those ocular manifestations. Given this, a baseline ophthalmic examination is recommended at diagnosis for all patients, with re-evaluation every 3 months. If optic nerve infiltration is present, more frequent follow-up is necessary, and any new visual symptoms should prompt immediate assessment. Long-term follow-up of patients in remission facilitates the timely detection and treatment of optic nerve involvement and CNS relapse. Clinicians should maintain a high index of suspicion for isolated optic neuropathy, especially in atypical cases of optic neuritis, when systemic findings are unremarkable. Early recognition and timely intervention can improve visual outcomes and may be life-saving by prolonging survival. Future collaborative prospective cohort studies between hematology/oncology and ophthalmology are needed to identify patients at high risk and to develop standardized treatment recommendations supported by evidence.

This study is limited by the small size of our case series and the largely case based, narrative nature of the available evidence. Reported diagnostic workup, treatments, and follow up are heterogeneous, which restricts conclusions about indications, timing, and long-term visual and survival outcomes for newer therapies. Larger multicenter studies with standardized data collection are needed to define optimal diagnostic algorithms, criteria for treatment escalation, and outcome predictors.

## Data Availability

The raw data supporting the conclusions of this article will be made available by the authors, without undue reservation.
